# Familial Risks Between Urolithiasis and Cancer

**DOI:** 10.1038/s41598-018-21410-0

**Published:** 2018-02-15

**Authors:** Kari Hemminki, Otto Hemminki, Asta Försti, Jan Sundquist, Kristina Sundquist, Xinjun Li

**Affiliations:** 1 Division of Molecular Genetic Epidemiology, German Cancer Research Center (DKFZ), Im Neuenheimer Feld 580, D-69120 Heidelberg, Germany; 2 Center for Primary Health Care Research, Lund University, 205 02 Malmö, Sweden; 3 Department of Urology, Helsinki University Hospital, Helsinki, Finland; 4 Cancer Gene Therapy Group, Faculty of Medicine, University of Helsinki, Helsinki, Finland; 5 Department of Family Medicine and Community Health, Department of Population Health Science and Policy, Icahn School of Medicine at Mount Sinai, New York, USA

## Abstract

Urolithiasis (UL, urinary tract stone disease) has been reported to increase subsequent cancers in the urinary tract. Recently, we showed data that surveillance bias may be an important confounder in the reported associations. In the present approach we want to address the question of possible cancer risk posed by UL mechanistically. Both UL and cancer have strong genetic components and we hypothesize that familial association between UL and cancer may be plausible. We thus assess familial risks between UL and cancer, hoping to find an explanation why UL may pose a risk of cancer. UL patients were identified from hospital inpatient and outpatient records and they were organized in families based on the Multigeneration Register into which also national cancer data were linked. Standardized incidence ratios were calculated for cancer in the offspring generation when parents were diagnosed with UL, and conversely for UL when parents were diagnosed with cancer. Familial risks between UL and cancer were generally small and inconsistent providing no convincing support of genetic sharing between UL and cancer. However, bladder UL was associated weakly with prostate cancer, and ureter and bladder UL were associated with salivary gland cancer. Potential mechanisms for these findings are proposed.

## Introduction

Urolithiasis (UL, urinary tract stone disease) includes stones found in the kidney, ureter and urinary bladder. UL is a common disease affecting up to 15% of population and many patients have recurrent episodes^1,2^. Kidney stones (nephrolithiasis) form in the kidney and leave the body in the urine stream. Small stones may pass without causing symptoms but stones measuring more than 5 mm tend to generate obstruction of the ureter causing severe pain. Some stones do not enter the ureter, instead they can grow to fill up the renal pelvis and cause kidney damage if untreated. Stones in the bladder have another etiology which usually relates to long term retention of urine in the bladder, typically through obstruction caused by prostate hyperplasia. Thus bladder stones are far more common among men than women. Bladder stones may form in the bladder but also seed on small stones originating from kidney with urine^1,2^. Reasons for UL are thought to be a combination of genetic and environmental factors. Risk factors include high urine calcium levels, calcium supplements, hyperparathyroidism, gout, obesity, dehydration, urinary stasis and some foods and medications. Genetic causes of UL include many rare monogenic metabolic disorders, such as adenine phosphoribosyltransferase deficiency, cystinuria, Dent disease, familial hypomagnesemia and primary hyperoxaluria^3^.

There are a number of papers on UL patients reporting subsequent risks of various cancers.

For example, a meta-analysis evaluated the association between personal history of kidney stones and kidney cancer, and collected results from 7 studies which gave an overall relative risk (RR) of 1.76, higher for transitional cell carcinoma than for renal cell carcinoma, and for renal cell carcinoma only men were at risk^4^. A Taiwanese case-control study on bladder stone patients found an RR of 3.42 for bladder cancer^5^. Another case-control study associate prostate cancer with prior kidney and bladder stones^6^. However, the reports are not only limited to urological cancers but a study from Taiwan’s National Health Insurance Research Database reported that UL was associated with a high risk of many systemic cancers, for example of breast and lung cancers (RRs 1.84 and 1.82)^7^.

We recently completed a study reconsidering the above results of UL’s possible role in subsequent cancers^8^. To our surprise the associations were strong with practically all cancers. However, they decreased with the length of the follow-up time since the last UL episode but for many cancers RRs remained significant even after 10 years of follow-up. We could not exclude that patients with recurrent UL disease might have contributed to the elevated risks after the long follow-up. We concluded that exclusion of surveillance bias is extremely difficult in conditions for which prior medical contacts have taken place^8^. As UL is a common disease it would be of high importance to unravel and settle the possible cancer risks because, if real, their population burden would be considerable and prevention would be at least in part possible. Here we decided to approach the problem through a mechanistic reasoning and addressing two research questions: 1) directly assessing whether UL and cancer share familiar links, and 2) indirectly using the results from 1) to conclude whether UL may be associated with individual cancer risk. As both UL and cancer have a genetic component we hypothesized that they share familial risks, i.e., in families with UL certain cancers should be in excess, and conversely, in families with cancer UL should be in excess. When UL and cancer would be assessed in different generations, surveillance bias should be non-existent or minimal. Demonstration of familial risk between UL and cancer would offer unbiased evidence and a plausible mechanism for individuals risks, i.e., why UL patients have an increased risk of cancer. We use the nation-wide Swedish Family-Cancer Database for the study.

## Results

Person numbers and characteristics of the UL and cancer patients are shown in Table 1 separately for the offspring (8.5 million) and parental (7.8 million) generations. Respective numbers of UL patients were 130,091 and 168,132, giving age-standardized incidence rates of 84.8 and 78.9 per 100,000. The numbers of cancer patients in the two generation were 529,923 and 474,686, and related incidence rates were 442.8 and 387.5 per 100,000. The relatively higher number of cancer patients was due to the longer follow-up period, starting from year 1958 for cancer and from year1987 for UL (for outpatients from years 2001). The median age at diagnosis was 8 years higher for cancer than for UL in the offspring generation but it was 17 years higher for the parental generation.

**Table 1:** Population and case numbers for urolithiasis and cancer.

	Offspring	Parents
Total population	8,468,901	7,759,522
Diagnosis of urolithiasis, 1987–2012	130,091	168,132
Kidney	50,139	61,479
Ureter	57,845	73,744
Mixed	16,269	18,926
Bladder	5,838	13,983
Mean age at diagnosis	47.0 ± 14.8	54.3 ± 16.3
Median age	48	54
Incidence rate per 100 000 person years*	84.8	78.9
Diagnosis of cancer, 1958–2012	529,923	474,686
Mean age at diagnosis	52.7 ± 15.2	69.2 ± 12.1
Median age	56	71
Incidence rate per 100 000 person years*	442.8	387.5

*Age adjusted for the European standard population.

A total of 33 different cancers were included in the analyses but we show data only for those cancers for which any association was significant, and for simplicity deleted data for the mixed UL type; however these data were included in the column for combined UL, ‘All’. In Table 2 standardized incidence ratios (SIRs) are shown for cancer in offspring when a parent was diagnosed with UL. The overall SIR was 1.02 (N = 15,729); note that case numbers were much lower than in Table 1 because only familial cases were included in Table 2 and subsequent Tables For 4 individual cancers SIRs were significant for all types of UL combined. The highest overall SIRs were observed for small intestinal cancer (1.42; 1.90 when parents had kidney stones) and salivary gland cancer (1.74; 1.97 when parents had ureter stones). Any significant association was observed for one UL type only. The overall SIR for prostate cancer was increased to 1.06 (95%CI 0.99–1.12) but no individual UL subtype showed an association (yet for bladder cancer in parents the SIR of 1.12 was of borderline significance, 95%CIs 0.99–1.25). There was no evidence on site specific concordance: urinary tract cancers were not increased by parental UL.

**Table 2:** SIR of cancer in offspring when parents were diagnosed with UL.

Cancer site in offspring	Kidney				Ureter				Bladder				All			
	O	SIR	95% CI		O	SIR	95% CI		O	SIR	95% CI		O	SIR	95% CI	
Upper aerodigestive tract	57	0.93	0.71	1.21	71	0.84	0.65	1.06	40	0.94	0.67	1.28	195	0.95	0.82	1.09
Salivary gland	13	1.48	0.78	2.53	23	**1.97**	**1.25**	**2.97**	7	1.52	0.60	3.16	48	**1.74**	**1.28**	**2.30**
Stomach	24	0.75	0.48	1.12	51	1.15	0.85	1.51	23	0.93	0.59	1.40	111	1.01	0.83	1.22
Small intestine	24	**1.90**	**1.21**	**2.82**	18	1.04	0.62	1.65	13	1.47	0.78	2.52	60	**1.42**	**1.08**	**1.83**
Colon	172	1.15	0.98	1.33	217	1.05	0.92	1.20	107	0.99	0.81	1.20	535	1.06	0.97	1.15
Rectum	81	0.90	0.71	1.12	132	1.05	0.88	1.24	68	0.98	0.76	1.24	305	0.99	0.88	1.10
Liver	88	**1.26**	**1.01**	**1.55**	86	0.88	0.70	1.09	50	0.97	0.72	1.28	254	1.06	0.94	1.20
Pancreas	31	0.89	0.61	1.27	55	1.11	0.83	1.44	28	0.97	0.64	1.40	125	1.02	0.85	1.21
Lung	127	1.05	0.88	1.25	148	0.87	0.73	1.02	90	0.87	0.70	1.07	399	0.93	0.84	1.03
Breast	665	1.05	0.98	1.14	903	1.05	0.98	1.12	450	1.06	0.96	1.16	2195	**1.05**	**1.01**	**1.09**
Cervix	1572	1.00	0.95	1.05	1978	0.98	0.94	1.03	437	0.94	0.85	1.03	4478	0.99	0.96	1.02
Endometrium	67	0.95	0.74	1.21	84	0.89	0.71	1.11	71	1.26	0.99	1.60	240	1.00	0.88	1.13
Prostate	289	1.08	0.96	1.21	384	0.98	0.89	1.09	292	1.12	0.99	1.25	1044	1.06	0.99	1.12
Kidney	57	1.02	0.78	1.33	78	1.06	0.83	1.32	31	0.85	0.58	1.21	188	1.04	0.89	1.20
Urinary bladder	68	1.05	0.82	1.34	96	1.06	0.86	1.29	44	0.82	0.59	1.10	221	0.98	0.85	1.11
Melanoma	404	1.07	0.97	1.18	533	1.06	0.98	1.16	219	**1.16**	**1.01**	**1.32**	1254	**1.07**	**1.01**	**1.13**
Nervous system	226	1.04	0.91	1.19	318	**1.14**	**1.02**	**1.27**	89	1.02	0.82	1.25	692	1.07	0.99	1.15
Endocrine glands	70	0.94	0.73	1.19	99	1.01	0.82	1.23	36	0.93	0.65	1.28	218	0.94	0.82	1.07
Bone	25	1.06	0.68	1.56	30	1.02	0.69	1.46	1	0.17	0.00	0.99	69	1.05	0.81	1.32
Hodgkin disease	56	1.03	0.78	1.34	67	0.99	0.77	1.26	14	1.01	0.55	1.70	157	1.03	0.88	1.21
Leukemia	126	0.93	0.77	1.10	172	1.00	0.85	1.16	53	0.94	0.70	1.23	389	0.96	0.87	1.06
Unspecified primary	52	0.98	0.73	1.29	74	1.01	0.80	1.27	34	0.85	0.59	1.19	171	0.95	0.81	1.10
All	5013	1.02	0.99	1.05	6642	1.02	0.99	1.04	2615	1.02	0.98	1.06	15729	**1.02**	**1.01**	**1.04**

Bold type: 95% CI does not include 1.00

O = observed number of cases; SIR = standardized incidence ratio; CI = confidence interval.

Column ‘All’ includes data for mixed stones which are not shown separately. They included 1459 cases for this table.

In Table 3, UL risk in offspring was assessed by parental cancer. The overall SIR was 1.04 (N = 74,361), and overall associations were significant with 14 parental cancers. Notably, only 4 overall associations reached an SIR of 1.10, these being with parental cancer in the salivary glands, endometrium, kidney and endocrine glands. We analyzed risks by specific endocrine glands, and the only significant SIR of 1.14 (N = 572, 1.05–1.23) for overall UL was with parathyroid gland tumors: among these, the only significant association of 1.25 was with ureter stones (N = 285, 1.11–1.40). Modest site specific concordance was observed for increased SIRs for kidney and ureter stones associated with kidney cancer. For bladder stones no association with bladder cancer was observed but the association of 1.15 with prostate cancer was significant. Overall UL associated with salivary gland cancers in Table 2 and Table 3. The highest individual associations were found for bladder stones: bone 2.19, Hodgkin lymphoma 1.82 and salivary glands 1.75.

**Table 3:** SIR of UL in offspring when parents were diagnosed with cancer.

Cancer site in parents	Kidney				Ureter				Bladder				All			
	O	SIR	95% CI		O	SIR	95% CI		O	SIR	95% CI		O	SIR	95% CI	
Upper aerodigestive tract	678	1.04	0.96	1.12	782	1.00	0.93	1.07	89	1.07	0.86	1.32	1759	1.02	0.97	1.07
Salivary gland	93	**1.28**	**1.03**	**1.57**	110	**1.25**	**1.03**	**1.50**	17	**1.75**	**1.02**	**2.81**	249	**1.28**	**1.13**	**1.45**
Stomach	932	0.97	0.91	1.03	1206	1.01	0.95	1.06	176	**1.16**	**1.00**	**1.35**	2649	1.01	0.97	1.05
Small intestine	125	0.99	0.82	1.18	176	1.15	0.99	1.34	15	0.94	0.53	1.56	353	1.05	0.95	1.17
Colon	2220	**1.07**	**1.02**	**1.11**	2745	**1.09**	**1.05**	**1.13**	303	**1.13**	**1.01**	**1.27**	5983	**1.08**	**1.06**	**1.11**
Rectum	1330	1.03	0.98	1.09	1672	**1.07**	**1.02**	**1.13**	177	1.07	0.92	1.24	3580	**1.05**	**1.01**	**1.08**
Liver	659	1.02	0.94	1.10	866	**1.09**	**1.02**	**1.17**	77	0.84	0.66	1.05	1811	**1.04**	**1.00**	**1.09**
Pancreas	686	1.06	0.98	1.14	803	1.01	0.94	1.09	105	1.16	0.95	1.40	1821	**1.05**	**1.00**	**1.10**
Lung	1949	**1.07**	**1.02**	**1.12**	2230	1.02	0.98	1.07	218	0.98	0.85	1.11	5006	**1.04**	**1.01**	**1.07**
Breast	3253	0.98	0.95	1.01	4053	**1.03**	**1.00**	**1.06**	377	0.96	0.87	1.06	8771	1.01	0.99	1.03
Cervix	3023	**1.05**	**1.01**	**1.08**	3256	1.02	0.98	1.05	313	**1.14**	**1.02**	**1.27**	7611	**1.04**	**1.02**	**1.06**
Endometrium	654	**1.16**	**1.07**	**1.25**	759	**1.10**	**1.02**	**1.18**	81	1.10	0.88	1.37	1686	**1.12**	**1.06**	**1.17**
Prostate	3300	0.99	0.96	1.02	4144	**1.03**	**1.00**	**1.06**	478	**1.15**	**1.05**	**1.25**	8999	**1.02**	**1.00**	**1.04**
Kidney	736	**1.13**	**1.05**	**1.21**	886	**1.11**	**1.04**	**1.19**	96	1.11	0.90	1.35	1970	**1.13**	**1.08**	**1.18**
Urinary bladder	1072	1.04	0.98	1.10	1326	**1.07**	**1.01**	**1.13**	137	1.05	0.88	1.24	2882	**1.06**	**1.02**	**1.10**
Melanoma	996	0.96	0.90	1.02	1221	1.02	0.96	1.08	111	1.06	0.87	1.27	2643	0.98	0.95	1.02
Nervous system	659	**1.12**	**1.03**	**1.21**	713	1.04	0.96	1.12	87	**1.28**	**1.03**	**1.58**	1674	**1.09**	**1.04**	**1.14**
Endocrine glands	327	1.08	0.96	1.20	425	**1.18**	**1.07**	**1.30**	33	0.92	0.63	1.30	889	**1.11**	**1.04**	**1.19**
Bone	40	0.99	0.71	1.35	50	1.07	0.79	1.40	10	**2.19**	**1.04**	**4.05**	118	1.12	0.93	1.35
Hodgkin disease	93	0.99	0.80	1.21	127	1.18	0.98	1.40	20	**1.82**	**1.11**	**2.81**	269	1.10	0.98	1.24
Leukemia	586	**1.10**	**1.01**	**1.19**	685	**1.08**	**1.00**	**1.16**	78	1.16	0.92	1.45	1533	**1.09**	**1.03**	**1.14**
Unspecified primary	581	**1.11**	**1.02**	**1.21**	625	0.98	0.91	1.06	70	1.00	0.78	1.26	1438	1.03	0.98	1.09
All	28031	**1.03**	**1.02**	**1.04**	33751	**1.04**	**1.03**	**1.05**	3548	**1.07**	**1.03**	**1.10**	74361	**1.04**	**1.03**	**1.05**

O = observed number of cases; SIR = standardized incidence ratio; CI = confidence interval.

Column ‘All’ includes data for mixed stones which are not shown separately. They included 9031 cases for this table.

Bold type: 95% CI does not include 1.00.

Table 4 shows risks of cancer in parents when offspring were diagnosed with UL. The analysis reversed offspring and parents as cases and probands from Table 2 and showed 5 times more cases. In agreement with Table 2, SIRs were increased for salivary gland and prostate cancers, and also for liver cancer, however the associated UL sites were different from Table 2.

**Table 4:** SIR of cancer in parents when offspring were diagnosed with UL.

Cancer site in parents	Kidney				Ureter				Bladder				All			
	O	SIR	95% CI		O	SIR	95% CI		O	SIR	95% CI		O	SIR	95% CI	
Upper aerodigestive tract	331	1.10	0.99	1.23	336	0.99	0.89	1.10	29	1.10	0.74	1.58	802	1.06	0.99	1.13
Salivary gland	36	1.29	0.91	1.79	37	1.17	0.82	1.61	5	1.98	0.63	4.66	91	**1.29**	**1.04**	**1.58**
Stomach	441	1.08	0.98	1.19	500	1.04	0.95	1.13	62	**1.42**	**1.09**	**1.82**	1163	**1.10**	**1.04**	**1.16**
Small intestine	79	1.13	0.90	1.41	83	1.04	0.83	1.29	7	1.08	0.43	2.24	182	1.02	0.88	1.18
Colon	1435	**1.08**	**1.02**	**1.13**	1683	**1.09**	**1.04**	**1.14**	141	1.09	0.92	1.29	3699	**1.08**	**1.05**	**1.12**
Rectum	800	1.04	0.97	1.12	941	1.06	0.99	1.13	80	1.10	0.87	1.37	2049	**1.04**	**1.00**	**1.09**
Liver	412	1.08	0.98	1.19	502	**1.13**	**1.03**	**1.23**	32	0.81	0.55	1.14	1067	**1.09**	**1.02**	**1.15**
Pancreas	407	1.08	0.98	1.19	448	1.03	0.93	1.13	44	1.18	0.86	1.59	1018	1.06	0.99	1.12
Lung	1291	**1.14**	**1.08**	**1.20**	1442	**1.11**	**1.05**	**1.17**	102	1.03	0.84	1.25	3233	**1.12**	**1.08**	**1.16**
Breast	1951	**0.93**	**0.89**	**0.97**	2298	0.98	0.94	1.02	159	0.90	0.76	1.05	5060	**0.96**	**0.93**	**0.98**
Cervix	609	**1.12**	**1.03**	**1.21**	555	1.06	0.98	1.15	44	1.15	0.84	1.55	1416	**1.11**	**1.05**	**1.16**
Endometrium	548	**1.09**	**1.00**	**1.18**	609	1.05	0.97	1.14	45	1.01	0.74	1.35	1376	**1.07**	**1.02**	**1.13**
Prostate	2763	1.01	0.97	1.05	3265	**1.04**	**1.01**	**1.08**	266	1.10	0.97	1.24	7164	**1.03**	**1.00**	**1.05**
Kidney	408	**1.13**	**1.02**	**1.24**	490	**1.18**	**1.08**	**1.29**	42	1.25	0.90	1.69	1074	**1.16**	**1.10**	**1.24**
Urinary bladder	810	1.06	0.98	1.13	915	1.03	0.97	1.10	67	0.94	0.73	1.19	2052	**1.05**	**1.00**	**1.09**
Melanoma	634	**0.91**	**0.84**	**0.98**	745	0.97	0.90	1.04	56	1.00	0.76	1.30	1632	**0.93**	**0.89**	**0.98**
Nervous system	393	**1.17**	**1.05**	**1.29**	400	1.08	0.98	1.19	36	1.33	0.93	1.84	947	**1.13**	**1.06**	**1.20**
Endocrine glands	237	1.03	0.90	1.17	279	1.08	0.96	1.22	22	1.09	0.68	1.65	606	1.04	0.96	1.13
Bone	15	1.02	0.57	1.68	17	1.05	0.61	1.69	1	0.82	0.00	4.70	40	1.09	0.78	1.48
Hodgkin disease	36	1.16	0.81	1.61	55	**1.62**	**1.22**	**2.11**	2	0.74	0.07	2.73	105	**1.36**	**1.11**	**1.64**
Leukemia	498	1.04	0.95	1.14	565	1.03	0.94	1.12	54	1.19	0.89	1.55	1265	1.04	0.98	1.09
Unspecified primary	614	**1.14**	**1.06**	**1.24**	692	**1.10**	**1.02**	**1.19**	63	1.16	0.89	1.49	1544	**1.12**	**1.06**	**1.17**
All	18181	**1.04**	**1.02**	**1.05**	20846	**1.04**	**1.03**	**1.06**	1701	**1.06**	**1.01**	**1.12**	46413	**1.04**	**1.03**	**1.05**

Bold type: 95% CI does not include 1.00.

O = observed number of cases; SIR = standardized incidence ratio; CI = confidence interval

Column ‘All’ includes data for mixed stones which are not shown separately. They included 5685 cases for this table.

In Table 5 gives SIRs for parental UL when offspring were diagnosed with cancers, thus reversing parents and offspring from Table 3; total case numbers decreased to 14,418. The two Tables shared overall increased association between UL and salivary gland and prostate cancers, and even the types of UL (ureter for salivary gland cancer and bladder for prostate cancer) were shared. Shared overall associations in Tables 5 and 3 were observed also for colon, cervical and nervous system cancers.

**Table 5:** SIR for UL in parents when offspring were diagnosed with cancer.

Cancer site in offspring	Kidney				Ureter				Bladder				All			
	O	SIR	95% CI		O	SIR	95% CI		O	SIR	95% CI		O	SIR	95% CI	
Upper aerodigestive tract	58	0.93	0.71	1.21	73	0.86	0.68	1.09	41	1.05	0.75	1.42	198	0.98	0.84	1.12
Salivary gland	14	1.55	0.85	2.61	23	**1.90**	**1.20**	**2.86**	6	1.34	0.48	2.95	48	**1.70**	**1.26**	**2.26**
Stomach	29	0.93	0.62	1.34	55	1.28	0.97	1.67	21	0.97	0.60	1.48	117	1.12	0.93	1.34
Small intestine	25	**1.90**	**1.23**	**2.81**	18	1.00	0.59	1.58	15	1.75	0.97	2.89	64	**1.47**	**1.13**	**1.88**
Colon	172	1.15	0.99	1.34	223	1.10	0.96	1.25	106	1.09	0.89	1.32	539	**1.10**	**1.01**	**1.20**
Rectum	90	0.92	0.74	1.13	145	1.08	0.91	1.27	66	0.96	0.74	1.22	326	0.99	0.89	1.11
Liver	45	1.33	0.97	1.78	41	0.90	0.65	1.23	26	1.18	0.77	1.74	123	1.11	0.93	1.33
Pancreas	29	0.84	0.56	1.20	52	1.09	0.81	1.43	26	1.03	0.67	1.50	118	1.01	0.83	1.21
Lung	121	1.10	0.91	1.31	137	0.91	0.76	1.07	87	1.08	0.87	1.33	377	1.01	0.91	1.12
Breast	608	1.05	0.97	1.14	856	**1.08**	**1.01**	**1.15**	423	**1.12**	**1.02**	**1.23**	2060	**1.08**	**1.03**	**1.13**
Cervix	1443	1.04	0.99	1.10	1830	1.04	0.99	1.09	381	0.98	0.88	1.08	4122	**1.05**	**1.02**	**1.08**
Endometrium	61	1.10	0.84	1.42	72	0.94	0.74	1.19	58	**1.40**	**1.06**	**1.80**	202	1.07	0.93	1.23
Prostate	260	1.06	0.94	1.20	361	1.05	0.95	1.17	260	**1.27**	**1.12**	**1.44**	948	**1.10**	**1.04**	**1.18**
Kidney	55	1.03	0.77	1.34	82	1.16	0.92	1.44	33	1.04	0.71	1.46	194	1.13	0.98	1.30
Urinary bladder	68	1.10	0.85	1.39	102	1.20	0.98	1.46	41	0.91	0.65	1.24	225	1.08	0.94	1.23
Melanoma	384	1.08	0.98	1.20	520	**1.10**	**1.01**	**1.20**	193	1.11	0.96	1.28	1195	**1.09**	**1.02**	**1.15**
Nervous system	211	1.11	0.97	1.27	278	**1.14**	**1.01**	**1.28**	65	0.88	0.68	1.13	608	**1.08**	**1.00**	**1.17**
Endocrine glands	67	0.98	0.76	1.25	106	1.18	0.96	1.42	36	1.07	0.75	1.48	222	1.05	0.92	1.20
Bone	21	1.00	0.62	1.53	34	1.28	0.89	1.79	1	0.19	0.00	1.11	70	1.18	0.92	1.50
Hodgkin disease	50	1.05	0.78	1.38	61	1.02	0.78	1.32	12	1.01	0.52	1.77	143	1.07	0.90	1.26
Leukemia	116	0.99	0.82	1.19	158	1.08	0.92	1.26	46	1.08	0.79	1.44	354	1.04	0.94	1.16
Unspecified primary	46	1.07	0.79	1.43	62	1.06	0.81	1.36	28	0.99	0.65	1.43	145	1.02	0.86	1.21
All	4589	**1.05**	**1.02**	**1.08**	6141	**1.07**	**1.04**	**1.09**	2330	**1.09**	**1.05**	**1.14**	14418	**1.07**	**1.05**	**1.09**

Bold type: 95% CI does not include 1.00.

O = observed number of cases; SIR = standardized incidence ratio; CI = confidence interval

Column ‘All’ includes data for mixed stones which are not shown separately. They included 1358 cases for this table.

## Discussion

Our goal was to investigate the possible familial association of UL and cancer^8^. If such an association could be demonstrated it would offer one possible mechanism for a personal history of UL being a risk factor for cancer. The demonstration of the association would require that positive results should be found in complementary two-way analyses, shown in Tables 2 to 5. Positive two-way results were found for salivary gland, liver and prostate cancers with UL. It is admitted that case numbers were very different in these Tables because parental cancer data spanned many more years than parental UL data. Early-onset cancers were relatively more presented in Table 2 compared to late-onset cancers. However, case numbers with 15,729 total cancers were quite large even in Table 2. Even in Table 3 with large case numbers only a few significant associations exceeded 1.10. However, for kidney cancer there were weak but concordant associations at anatomic sites with kidney, ureter and mixed UL in Table 3, which may signal biological plausibility. In order to confirm that associations were not only due to the disparate follow-up times additional comparisons between offspring and parents were carried out (Tables 4 and 5). These essentially replicated the results for salivary gland and prostate cancers, and gave some further support to the possible association of kidney cancer with mixed UL. In addition to disparate follow-up times, other limitations of the study were lacking information on the types of stones and on comorbidities. The results were largely negative and, with the exception of salivary gland, liver and prostate cancers, no familial associations were found with UL and cancer, even when considering site specific cancers in the urinary tract. Thus the overall conclusion was that there was no overall familial association between UL and cancer.

The present incidence of UL was about 80/100,000 in the parental and offspring generations. Surprisingly, incidence rates of UL are rarely reported in the global literature even in studies which refer to incidence in the title; if at all, these may give incidence in a defined subpopulation. However, population based incidence rates for renal stones (including kidney and ureter) were 100.1/100,000 in Rochester, US in year 2000^9^. The present incidence rates for cancer (400/100,000) were at the level of the Swedish rates in the 1990s^10^.

The associations of UL with prostate cancer were not strong but they were most consistent with bladder UL. The reason could be shared familial risk but there is an alternative explanation. Prostate cancer has a large familial component whereby fathers and sons may both have prostate cancer^11^. Prostate hyperplasia is a risk factor for diagnosis of prostate cancer, and is also known to be familial; it is thus likely that both fathers and sons suffer from this condition^12,13^. The alternative explanation would then be familial tendency for bladder outlet obstruction and the resulting urine retention as a course of bladder stones.

The most consistent familial associations between cancer and UL were found with salivary gland cancers and ureter stones, but also with kidney and bladder stones. Salivary glands and particularly the submandibular gland may harbor stones in their ductal systems, referred to as sialolithiasis^14^. X-ray microanalysis has shown that sialoliths and kidney stones have largely similar elemental composition of calcium, phosphorous, magnesium, sodium, chloride, silicon, iron, and potassium^14^. A further clue to the puzzle is a case-control study on close to 1000 sialolithiasis patients identified from the Taiwan Longitudinal Health Insurance Database^15^. A significant difference in the prevalence of prior nephrolithiasis was found between cases and controls (10.25% vs 2.28%, p < 0.001) with a relative risk of 4.74^15^. We searched literature and consulted an expert on the possible relationship between sialolithiasis and salivary gland cancer but found no relevant data. Hypothetically, if sialolithiasis were related to salivary gland cancer the present results would make sense as sialolithiasis and UL are associated with each other. Thus the present link between UL and salivary gland cancer would be explained by sialolithiasis associating with salivary gland cancer.

The association of parathyroid tumors with ureter stones may point to a mechanistic basis because parathyroid tumors are usually diagnosed because of hypercalcemia and hypercalciuria and these conditions are important risk factors for UL^1,16^. However the risk was modest and the SIR reached statistical significance only in Table 3.

In conclusion, the present study did not provide data in support of UL leading to systemic cancers. Nor did we find any strong support for the induction of local tumors in the urinary tract; however, as the cause for local tumors may be chronic mechanical wear and inflammation, a family study may not find such a link^4,5,7^. We found support for a weak familial association of bladder UL and prostate cancer but could not distinguish between genetic or familial prostate hyperplasia mechanisms. Unexpected findings showed associations of ureter and bladder UL with salivary gland cancers. The likely initial link was familial predisposition to both salivary gland and urinary tract stones but the necessary final link between salivary gland stones and salivary gland cancer needs yet to be demonstrated.

## Patients and Methods

Family relationships were obtained from the Multigeneration Register, containing the Swedish population in families and spanning more than a century. ‘The offspring generation’ was born after 1931 and ‘the parental generation’ was born any time earlier. By the last year of the study, 2012, the offspring generation reached age 80 years. The offspring generation with information of both parents totaled 8.5 million index individuals. UL patients were identified using the nationwide Swedish Hospital Discharge Register (1987–2012) and the Outpatient Register (2001–2012). The first UL diagnosis in either register was included and a patient was only entered once. Information from the registers was linked at the individual level via the national 10-digit civic registration number to the Swedish national Cancer Registry. Both invasive and *in situ* cancers were included; however *in situ* cases contributed essentially only to cervical cancer. In the linked dataset, civic registration numbers were replaced with serial numbers to ensure the anonymity. Revisions 9 (1987–1996) and 10 (1997-) of the International Classification of Diseases (ICD) was used to identify UL diagnostic codes. Only 54,500 patients were diagnosed during the ICD-9 period, compared to 166,600 in the ICD-10 period. The total number of patients diagnosed with UL during years 1987 to 2012 was 211,718, distributed by the most common type, ureter stones (91,397), followed by kidney stones (77,972), mixed stones (23,890) and bladder stones (18,459). For mixed stones the location between kidney and ureter was undefined or stones were present in both.

Standardized incidence ratios (SIRs) were calculated for the offspring generation as the ratio of observed to expected number of cases. SIRs were calculated for cancer in offspring whose parents were diagnosed with UL, or conversely, for UL in offspring whose parents were diagnosed with cancer. The follow-up for cancer was started from January 1^st^ 1958, date of birth or date of immigration whichever came last, and continued until diagnosis of cancer, death, emigration, or the end of the study (December 31^st^, 2012) whichever came first. Follow-up for UL was started from 1987 and ended at diagnosis of UL, death or end of follow-up, 2012. The expected numbers were calculated for all individuals without a family history of UL or of cancer (essentially the whole Swedish population), and the rates were standardized by 5-year-age, gender, period (5 years group), socioeconomic status (farmers, self-employed, professionals, white collar workers, blue collar workers, others) and residential area (large cities, southern Sweden, northern Sweden). The 95% confidence interval (95%CI) of the SIR was calculated assuming a Poisson distribution. The SAS software version 9.3 was used for the statistical analyses.

The study was approved by the Regional Ethical Review Board of Lund University (no. 2012/795). The ethical permission waived informed consent because anonymous health records were used. The study was conducted following relevant regulations.

## References

[1] Tiselius HG (2003). Epidemiology and medical management of stone disease. BJU Int.

[2] Morgan MS, Pearle MS (2016). Medical management of renal stones. Bmj.

[3] Edvardsson VO (2013). Hereditary causes of kidney stones and chronic kidney disease. Pediatric nephrology (Berlin, Germany).

[4] Cheungpasitporn W (2015). The risk of kidney cancer in patients with kidney stones: a systematic review and meta-analysis. QJM: monthly journal of the Association of Physicians.

[5] Chung SD, Tsai MC, Lin CC, Lin HC (2013). A case-control study on the association between bladder cancer and prior bladder calculus. BMC Cancer.

[6] Chung SD, Liu SP, Lin HC (2013). Association between prostate cancer and urinary calculi: a population-based study. PLoS One.

[7] Shih CJ (2014). Urinary calculi and risk of cancer: a nationwide population-based study. Medicine.

[8] Hemminki K (2017). Surveillance bias in cancer risk after unrelated medical conditions: example urolithiasis. Scientific reports.

[9] Lieske JC (2006). Renal stone epidemiology in Rochester, Minnesota: an update. Kidney Int.

[10] CenterforEpidemiology. Cancer Incidence in Sweden 2009. (ed^eds). The National Board of Health and Welfare (2010).

[11] Frank C, Sundquist J, Hemminki A, Hemminki K (2017). Familial Associations Between Prostate Cancer and Other Cancers. Eur Urol.

[12] Stroup SP, Palazzi-Churas K, Kopp RP, Parsons JK (2012). Trends in adverse events of benign prostatic hyperplasia (BPH) in the USA, 1998 to 2008. BJU Int.

[13] Orsted DD, Bojesen SE (2013). The link between benign prostatic hyperplasia and prostate cancer. Nature reviews Urology.

[14] Rakesh N, Bhoomareddy Kantharaj YD, Agarwal M, Agarwal K (2014). Ultrastructural and elemental analysis of sialoliths and their comparison with nephroliths. Journal of investigative and clinical dentistry.

[15] Wu CC (2016). Sialolithiasis is associated with nephrolithiasis: a case-control study. Acta oto-laryngologica.

[16] DeLellis, R., Lloyd, R., Heitz, P. & Eng, C. Pathology & Genetics of Tumours of Endocrine Origin. In: *World Health Organization Classification of Tumours* (ed^eds). IARC Press, Lyon (2004).

